# Inflammation Regulates TMPRSS6 Expression via STAT5

**DOI:** 10.1371/journal.pone.0082127

**Published:** 2013-12-23

**Authors:** Delphine Meynard, Chia Chi Sun, Qifang Wu, Wenjie Chen, Shanzhuo Chen, Caroline N. Nelson, Michael J. Waters, Jodie L. Babitt, Herbert Y. Lin

**Affiliations:** 1 Program in Anemia Signaling Research, Division of Nephrology, Program in Membrane Biology, Center for Systems Biology, Massachusetts General Hospital, Harvard Medical School, Boston, Massachusetts, United States of America; 2 Institute for Molecular Bioscience, University of Queensland, St. Lucia, Australia; Baylor College of Medicine, United States of America

## Abstract

*TMPRSS6* is a regulated gene, with a crucial role in the regulation of iron homeostasis by inhibiting hepcidin expression. The main regulator of iron homeostasis, the antimicrobial peptide hepcidin, which also has a role in immunity, is directly upregulated by inflammation. In this study, we analyzed whether inflammation is also a modulator of *TMPRSS6* expression *in vitro* and *in vivo* and we determined the mechanism of this regulation A Human Hepatoma cell line was treated with interleukin-6 and mice were injected with lipopolysaccharide and TMPRSS6 expression and the regulatory mechanism were addressed. In this study, we demonstrate that inflammation downregulates *TMPRSS6* expression *in vitro* and *in vivo*. The downregulation of Tmprss6 by inflammation in mice is not dependent on the Bmp-Smad pathway but occurs through a decrease in Stat5 phosphorylation. Moreover, Stat5 positively regulates Tmprss6 expression directly by binding to a Stat5 element located on the Tmprss6 promoter. Importantly, our results highlight the functional role of inflammatory modulation of *TMPRSS6* expression in the regulation of hepcidin. TMPRSS6 inhibition via decreased STAT5 phosphorylation may be an additional mechanism by which inflammation stimulates hepcidin expression to regulate iron homeostasis and immunity.

## Introduction

The transmembrane serine protease matriptase-2, encoded by the *TMPRSS6* gene, is expressed in the liver and is a negative regulator of hepcidin expression [1]. In humans, mutations in *TMPRSS6* lead to a genetic disorder characterized by an iron refractory iron deficiency anemia (IRIDA) that is unresponsive to oral iron treatment but partially responsive to parenteral iron therapy [2]. The importance of *TMPRSS6* in the control of iron homeostasis and normal erythropoiesis in humans has been highlighted by genome-wide association studies. These studies identified common *TMPRSS6* variants associated with hematological parameters and serum iron concentration [3].

Hepcidin is a peptide hormone produced by the liver that controls iron absorption at the intestinal level, and iron release from macrophages and hepatocytes. Hepcidin binds to the plasma membrane iron exporter ferroportin and induces its endocytosis and proteolysis, preventing release of iron into the plasma [4]. It is now well established that hepcidin expression is regulated by the BMP-SMAD pathway in response to iron variation [5]. Mutated forms of matriptase-2 are unable to cleave membrane hemojuvelin [6], resulting in a stimulation of the BMP-SMAD signaling pathway and an inappropriately high hepcidin expression.

Recently, matriptase-2 has been demonstrated to be induced by acute iron deprivation [7], hypoxia [8], and erythropoietin [9], and by activators of hepcidin expression such as BMP6 and iron [10]. It is likely that matriptase-2 is upregulated by these activators of hepcidin expression as a negative feedback mechanism to control excessive increases in hepcidin. Thus, matriptase-2 has a pleiotropic role in hepcidin regulation in response to a number of stimuli.

Inflammation is a potent stimulator of hepcidin expression. The upregulation of hepcidin in response to inflammation promotes hypoferremia through the downregulation of ferroportin iron export activity. Hepcidin induction has been hypothesized to have a protective role in infection by sequestering iron from invading pathogens. As an antimicrobial peptide, hepcidin itself may also have additional roles in immunity [11].

IL-6 is a major hepatic regulator of the acute-phase response to inflammatory stimuli including hepcidin induction [12]. IL-6 binding to the IL-6 receptor leads to activation of Janus kinases that phosphorylate STAT3. Translocation of STAT3 to the nucleus results in upregulation of hepcidin expression through STAT3 responsive element on the hepcidin promoter [13]. In response to inflammatory stimuli, the BMP-SMAD pathway is also required to activate hepcidin [14], which may involve the activation of activin B [15].

Although hepcidin expression is induced directly by inflammatory stimuli, we hypothesized that additional fine tuning of its expression may be required to maintain body iron balance through the regulation of other genes. Specifically, we hypothesized that *TMPRSS6* expression could be regulated by inflammation in order to participate in the regulation of hepcidin. In this study, we show that *TMPRSS6* expression is regulated by inflammation via STAT5.

## Methods

### Cell Culture

Hep3B cells (HB-8064, ATCC, Manassas, VA) were cultured in ATCC-formulated EMEM (ATCC) supplemented with 10% fetal bovine serum (FBS, ATCC). Hepa1–6 cells (CRL-1830, ATCC) were cultured in ATCC-formulated DMEM (ATCC) supplemented with 10% fetal bovine serum (FBS, ATCC).

### Treatment of Hep3B Cells with IL-6

Hep3B cells (1.2×10^5^ per well) were seeded onto 24-well plates. Twenty-four hours later, the culture medium was switched to 1% FBS medium. After 7 hours, cells were treated with recombinant human IL-6 (5, 20, 100 ng/mL, 16 h) (R&D Systems) and then harvested for RNA extraction.

Regarding matriptase-2 activity, after 7 hours, culture medium was replaced with Optimem media (Invitrogen, Carlsbad, CA) and cells were treated with recombinant human IL-6 (20 ng/mL) for 16 hours. Matriptase-2 activity was assayed as previously described [10].

### Animals

The Institutional Animal Care and Use Committee at the Massachusetts General Hospital (MGH) approved all of the following animal protocols.

Eight-week-old male C57BL/6 mice received four intraperitoneal injections of recombinant mouse IL-6 (406-ML, R&D systems, Minneapolis, MN) in PBS at 25 µg/k (one injection every 3 hours). (N = 5 per group). Mice were sacrificed and tissues harvested for analysis three hours after the last injection.

For LPS experiments, 8-week-old C57BL/6 males (Taconic Germantown, NY) received an intraperitoneal injection of LPS diluted in PBS at 1 µg/g body weight (serotype 055:B5, Sigma, Allentown, PA) (n = 5 per group). Mice were sacrificed and tissues harvested for analysis at 6, 16, and 24 hours after injection.

8-week-old males *Hjv* ^−/−^ mice on a C57BL/6J background, or wild-type littermate received an intraperitoneal injection of LPS diluted in PBS at 1 µg/g body weight (Sigma) (n = 5 per group). Mice were sacrificed and tissues harvested for analysis 6 hours after injection.

### RNA Extraction and Quantitative Real-time PCR

Total RNA and quantitative real-time RT-PCR were prepared as previously described [10]. *TMPRSS6*, *HAMP* and *RPL19* transcripts were amplified with specific primers (Table S1).

### Western-blot Analysis

Nuclear proteins were extracted from mice livers with NE-PER Nuclear and Cytoplasmic Extraction Reagents (Thermo Scientific, Rockford, IL). Equal amounts of protein were subjected to SDS-PAGE and transferred to PVDF Membrane (Biorad). For p-SMAD1-5-8, membranes were blocked with stringent milk buffer [5] 1 hour RT and then incubated overnight 4°C with rabbit anti-Phospho-Smad1 (Ser463/465)/Smad5 (Ser463/465)/Smad8 (Ser426/428) (1∶500, Cell Signaling, Beverly, MA). For p-Stat5, membranes were incubated in blocking buffer (5% milk, 0.1% Tween20, 1×PBS), 1 hour RT and then incubated overnight 4°C with rabbit anti-Phospho-Stat5 (Tyr694) (1∶1000, Cell Signaling). All blots were washed with PBST (0.1% Tween20, 1×PBS) and then incubated for 1 hour at RT with Anti-rabbit IgG, HRP-linked Antibody (1∶2000, Cell Signaling) in 5% milk PBST and developed using a chemiluminescence (ECL, PerkinElmer, Waltham, MA). Protein loading was controlled with Anti-TBP antibody. Chemiluminescence was quantified with IPLab imaging software (BD Biosciences).

### Electrophoretic Mobility Shift Assay (EMSA)

The Li-Cor EMSA buffer kit (Li-Cor, Lincoln, NE) was used according to the manufacturer’s instructions. Infrared (IR) labeled custom oligos specifics for the STAT5 element from mouse Tmprss6 promoter were ordered from Integrated DNA Technologies (Coralville, IA).

A total of 5 µg of nuclear protein extract was incubated with 2.5 nM of labeled oligos, 1× binding buffer, Poly (dl·dC) 1 µg/µL (in 10 mM Tris, 1 mM EDTA, pH 7.5), 25 mM DTT/2.5% Tween-20, 1% NP-40, 100 mM MgCl2, and 4% Ficoll for 30 minutes at room temperature shielded from light. DNA/protein complexes were visualized on a native 4–12% Tris-Borate-EDTA polyacrylamide gel (Invitrogen). Gels were immediately removed from cassettes and scanned using the Odyssey.

For WEMSA experiments, after EMSA, nucleic acids and bound proteins were transferred onto nitrocellulose membrane (Invitrogen). Blots were blocked with 5% milk PBST 1 hour RT, and incubated overnight at 4°C with rabbit anti-STAT5 antibody (1∶500, Santa-Cruz Biotechnology, Santa-Cruz, CA) diluted in 5% milk PBST. After washing with PBST 0.1%, blots were incubated for 1 hour at RT with Anti-rabbit IgG, HRP-linked Antibody (1∶2000, Cell Signaling) in 5% milk PBST and developed using a chemiluminescence (ECL, PerkinElmer).

### CHIP Experiments

Chromatin immunoprecipitation was performed on the liver of 8-week-old C57BL/6 males (baseline) using SimpleChIP Plus Enzymatic Chromatin IP Kit (Cell Signaling) according to the manufacturer’s instructions. Briefly, tissues were fixed with 1.5% formaldehyde for 20 minutes. DNA was sonicated using a Misonix XL Sonicator Ultrasonic Cell Processor (power, 40%; 3×20 seconds). DNA fragments were recovered using rabbit anti-Stat5 (Santa-Cruz Biotechnology) or normal rabbit anti-IgG. Recovered DNA fragments were directly used for quantitative real-time PCR analysis with specific primers (Table S1) for the region of the mouse Tmprss6 promoter containing the STAT5 element.

### SiRNA Experiments

Hep3B cells (1.2×10^5^ per well) were seeded onto 24-well plates. During seeding, cells were reverse-transfected with 1 µl of lipofectamine 2000 (Invitrogen) and siRNA control (ON-TARGET plus Non-targeting pool D-001810-10-05, Dharmacon, Chicago, IL) or human STAT5b (siGENOME SMART pool siRNA M-010539-02-0005, Dharmacon) or human TMPRSS6 (siGENOME SMART pool siRNA D-006052, Dharmacon). Five hours later, the transfection media was replaced with culture medium to stop the transfection.

For STAT5 silencing, 24 hours later, cells were serum starved with FBS 1% medium then harvested for RNA extraction 24 hours later.

For TMPRSS6 silencing, 24 hours later, cells were serum starved with FBS 1% medium and 7 hours later treated with IL-6 (20 ng/mL, R&D Systems) and harvested for RNA extraction 48 hours after treatment.

### Luciferase Assay

Mouse *Tmprss6* promoter was generated by amplifying 300 ng mouse genomic DNA (C57BL/6) using primers listed in Table S1. The resulting PCR fragment was inserted into the pGl3-promoter vector (Promega, Madison, WI). The STAT5 mutation construct was generated with QuikChange Site-Directed Mutagenesis Kit (Stratagene, La Jolla, CA, USA) using the primers presented in Table S1. Hepa 1–6 cells (1.2×10^5^ per well) were seeded onto 24-well plates and transfected 24 hours later for 5 hours with mouse *Tmprss6* wt-luc vector or mouse *Tmprss6* mt-luc vector (250 ng), pRL-TK (25 ng) and 200 ng of pcDNA empty vector or mouse *Stat5b* vector with 2 µl of lipofectamine 2000 (Invitrogen) in Optimem media. After 24 hours, cells were serum starved with 1% FBS media for 24 hours and cell lysates were prepared with 1X passive lysis buffer (Promega). Luciferase activity was determined with Dual-Luciferase® Reporter Assay System (Promega) according to manufacturer’s directions. The assay was performed in duplicate for each experiment. Our results were reported as RLU (relative light units).

### Statistics

For each quantitative variable (mRNA expression, protein level, matriptase 2 activity, luciferase activity), individual values were divided by a calibrator quantity, which was the mean value of this variable for the control group (baseline, mock, control). Means+/− SEM of the calibrated values obtained within each group are shown on the different figures. Calibrated values in two groups (control/treated) were compared by Student t tests.

**Figure 1:**
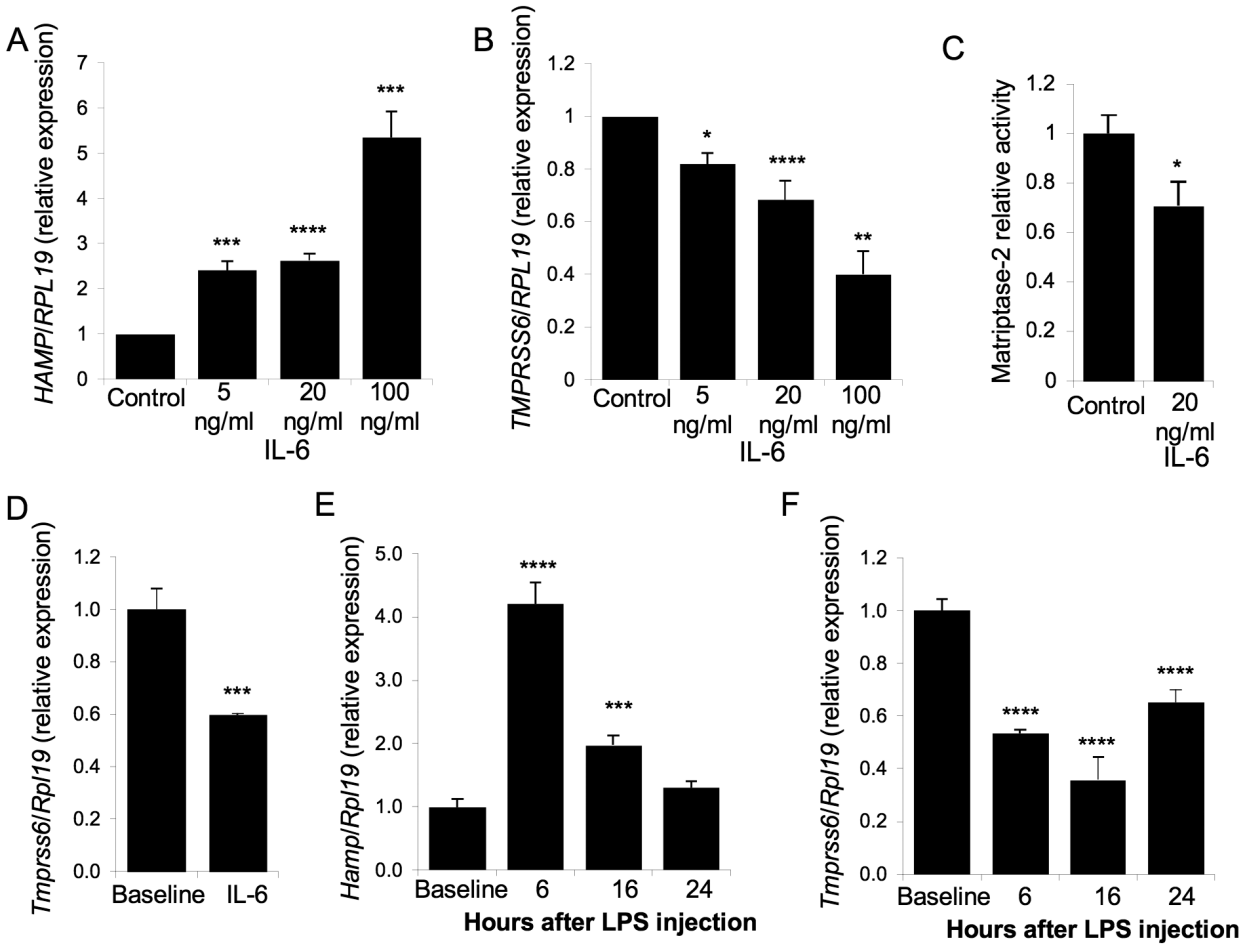
*Tmprss6* expression is down-regulated by inflammation. (A–B) Hep3B cells were treated with IL-6 for 16 hours and were analyzed for *HAMP* and *TMPRSS6* relative to *RPL19* mRNA expression. For each experiment, raw data were normalized to the value of the non-treated group. Values shown are means of normalized expression values for 4–7experiments+/− SEM. Means in treated groups were compared to 1 by one sample student t tests. (C) 15 µg of protein from conditioned media of Hep3B cells treated with IL-6 (20 ng/mL) for 16 hours were used to measure the matriptase-2 activity. Activities were measured in duplicate in 4 independent experiments. Values shown are mean of activities divided by a activities calibrator quantity (the mean value of activity for the control group)+/− SEM. Mean in control and treated groups were compared by student t tests. (D) Mice received 4 intraperitoneal injections of recombinant mouse IL-6 and were sacrificed 3 hours after the last injection. *Tmprss6* relative to *Rpl19* mRNA expression was analyzed by quantitative real-time RT- PCR. Values shown are means of expression values divided by a calibrator quantity (the mean value of expression for the baseline group)+/− SEM. Means in baseline and treated groups were compared by student t tests. (E–F) Mice received one injection of LPS (n = 5 per group) and were sacrificed 6, 16 and 24 hours after injection. *Hamp* and *Tmprss6* relative to *Rpl19* mRNA expression were analyzed by quantitative real-time RT- PCR. Values shown are means of expression values divided by a calibrator quantity (the mean value of expression for the baseline group)+/− SEM. Means in baseline and treated groups were compared by student t tests. p values p<0.05 were considered statistically significant. Significances are: *p<0.05; **p<0.01; ***p<0.005; ****p<0.001.

**Figure 4:**
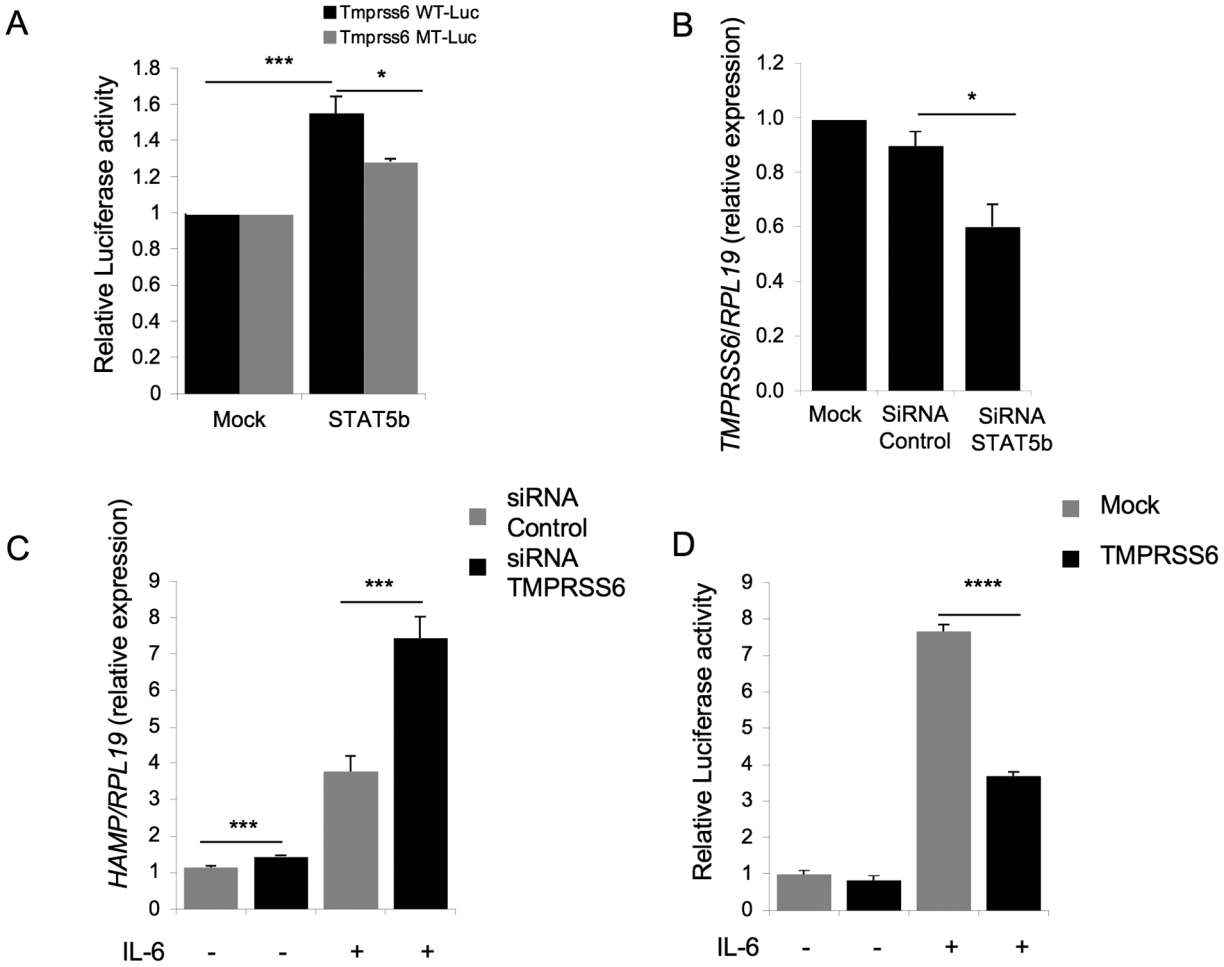
Stat5 regulates Tmprss6 expression and this participates to the regulation of hepcidin expression by IL-6 treatment. (A) Hepa 1–6 cells were transfected with mouse wild-type or mutant *Tmprss6* promoter-luciferase construct, in presence of empty vector or Stat5b plasmid. After 48 hours, luciferase activity was determined. The assay was performed in duplicate for a total of 6 independent experiments. For each experiment, raw data were normalized to the value of luciferase activity in cells transfected with the empty vector. Values shown are mean of normalized luciferase activities+/− SEM. Mean in treated groups was compared to 1 by one sample student t tests. (B) Hep3B cells were transfected with siRNA control or siRNA Stat5b and analyzed for *TMPRSS6* relative to *RPLl19* mRNA expression. For each experiment, raw data were normalized to the expression value in mock-treated cells. Values shown are means of normalized expression values obtained in 3 independent experiments+/− SEM. Means in siRNA control and siRNA STAT5b groups were compared by student t tests. (C) Hep3B cells were transfected with siRNA control or siRNA TMPRSS6 and treated without or with 20 ng/mL of IL-6, followed by analysis of *HAMP* relative to *RPL19* mRNA expression. The mean 4 independent experiments is presented. Values shown are means of expression values divided by a calibrator quantity (the mean value of expression for the cells transfected with siRNA control non-treated with IL-6)+/− SEM. Means in non-treated and IL-6 treated groups were compared by student t tests. (D) Hep3B cells were transfected with human *HAMP* promoter-luciferase construct in combination with either empty vector or human *TMPRSS6* plasmid. Thirty-two hours after transfection, cells were treated without or with 20 ng/ml of IL-6. After 16 hours of IL-6 treatment, luciferase activity was determined. The assay was performed in duplicate for a total of 6 independent experiments. Values shown are means of luciferase activity divided by a calibrator quantity (the mean value of luminescence for the mock non-treated with IL-6)+/− SEM. Means in non-treated and IL-6 treated groups were compared by student t tests. P values p<0.05 were considered statistically significant. Significances are: *p<0.05.

For experiments figures 1A, 1B, 4A, 4B, S1 and S6, raw data were normalized to the value of the non-treated control. Means in treated groups were compared to 1 by one sample student t tests.

## Results

### *TMPRSS6* mRNA Expression is Decreased Acutely by IL-6 Treatment *in vitro*

To test whether IL-6 treatment could modulate *TMPRSS6* mRNA expression *in vitro*, human hepatoma-derived Hep3B cells were treated with increasing doses of IL-6, and then *HAMP* and *TMPRSS6* mRNA expression was evaluated by quantitative real-time PCR. Treatment with IL-6 induced a dose-dependent increase of *HAMP* mRNA expression (from 2.4 with 5 ng/mL to 5.3-fold with 100 ng/mL) (Figure 1A). In contrast, *TMPRSS6* mRNA was significantly decreased by IL-6 treatment in a dose dependent manner (from 18% with 5 ng/mL to 60% with 100 ng/mL) (Figure 1B). The ability of IL-6 to decrease TMPRSS6 expression was also demonstrated in another human hepatoma-derived cell line, HepG2 cells (Figure S1A–B).

In order to validate that the decrease in *TMPRSS6* mRNA expression induced by IL-6 treatment was functionally relevant, we measured matriptase-2 activity in conditioned media derived from Hep3B cells treated with IL-6. Treatment with 20 ng/mL of IL-6 lowered matriptase-2 activity by 30% in conditioned media (Figure 1C). The specificity of the assay for matriptase-2 protease activity was previously demonstrated [10]. These results indicate that *in vitro*, IL-6 downregulates *TMPRSS6* mRNA expression and leads to a decrease of matriptase-2 activity.

### *TMPRSS6* mRNA Expression is Decreased Acutely by Inflammation *in vivo*

Next, we investigated whether Il-6 could regulate *Tmprss6* mRNA expression *in vivo*. Mice received four injections (every three hours) of 25 µg/kg of Il-6 and hepatic *Tmprss6* mRNA expression was measured 3 hours after the last injection. Compared to baseline mice, Il-6 injected-mice had a significant downregulation of liver *Tmprss6* mRNA level by 40% (Figure 1D). This result shows that the downregulation of *Tmprss6* mRNA expression by IL-6 seen *in vitro* also occurs *in vivo*.

Since injection of IL-6 had just a short-term effect on*Tmprss6* mRNA expression *in vivo*, possibly related to rapid clearance of injected Il-6, we tested whether *Tmprss6* mRNA expression was modulated in an inflammation model where IL-6 was highly induced. Mice were injected with 1 µg/g body weight of lipopolysaccharide (LPS), a potent inducer of inflammation [16]. The presence of an inflammatory state was established by the measurement of acute-phase genes *Crp* and *Il-6* in LPS-treated mice (Figure S2). LPS injection lead to a significant increase in hepatic *Hamp* mRNA by 4-fold at 6 hours after injection compared to baseline mice, returning to the basal level 24 hours after injection (Figure 1E). Importantly, as partially reported during the preparation of this manuscript [17], LPS injection significantly decreased *Tmprss6* mRNA expression with a maximal reduction of 64% at 16 hours after injection (Figure 1F) compared to baseline mice. These results indicate that *Tmprss6* mRNA expression is decreased acutely by inflammation *in vivo,* at least in part via Il-6.

### Inflammation Regulates *Tmprss6* mRNA Expression Through Stat5

**Figure 2:**
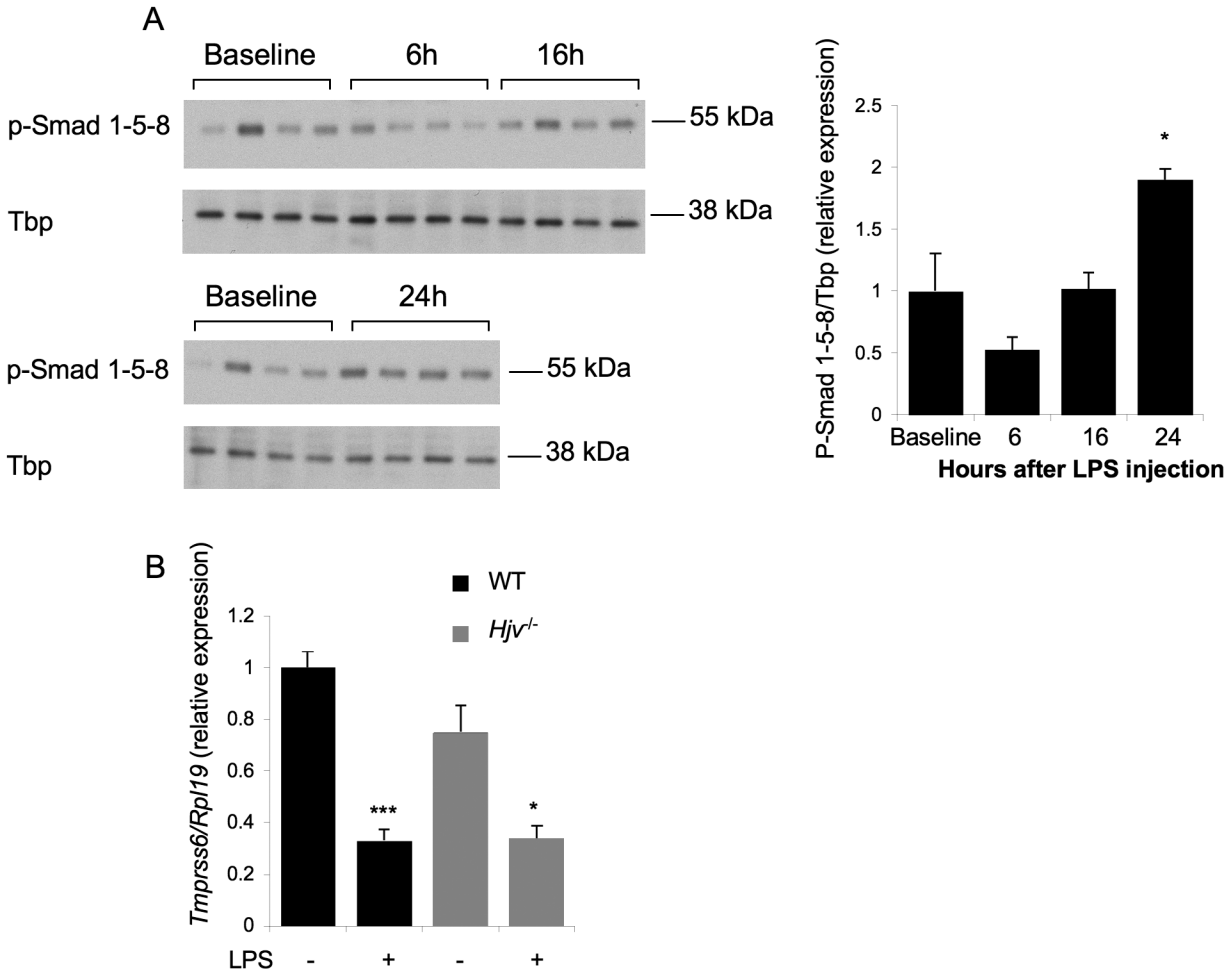
TMPRSS6 downregulation by inflammation is not dependent on the BMP-SMAD pathway. (A) Liver nucleic lysates from mice were analyzed by Western blot for p-Smad1-5-8 relative to TBP. Values shown are means of expression values divided by a calibrator quantity (the mean value of expression for the baseline group)+/− SEM. Means in baseline and treated groups were compared by student t tests. (B) WT and *Hjv* ^−/−^ littermate mice were injected with LPS (n = 4 per group) and sacrificed 6 hours later. *Tmprss6* relative to *Rpl1 9* mRNA expression was analyzed by quantitative real-time RT- PCR. Values shown are means of expression values divided by a calibrator quantity (the mean value of expression for the mock group)+/− SEM. Means in mock and treated groups were compared by student t tests. P values p<0.05 were considered statistically significant. Significances are: *p<0.05; ***p<0.005.

Because *Tmprss6* mRNA expression is regulated by the Bmp-Smad pathway [10], we investigated whether the Bmp-Smad pathway was also involved in the regulation of *Tmprss6* mRNA expression by inflammation. In wild-type mice, injection of LPS induced a trend toward decreased phosphorylation of Smad1-5-8 protein by 50% 6 hours after injection, and a return to baseline levels 16 hours after injection (Figure 2A). These results suggest only a partial temporal correlation between Bmp-Smad pathway activity and *Tmprss6* mRNA regulation by inflammation. To further explore a causative role for the BMP-SMAD pathway, we injected LPS into *Hjv^−/−^* mice, where the Bmp-Smad pathway is inhibited [18]. Similar to the results obtained in wild-type littermate mice, injection of LPS in *Hjv^−/−^* mice lead to a similar downregulation of *Tmprss6* mRNA expression by 57%, indicating that an intact Bmp-Smad pathway is not required for the regulation of *Tmprss6* expression by inflammation (Figure 2B).

Because inflammation modulates *Hamp* expression via the phosphorylation of Stat3 we investigated the regulation of p-Stat3 in the liver in response to LPS injection. Six hours after LPS injection, Stat3 phosphorylation was strongly increased and then progressively returned to baseline (Figure S3). These results show that the regulatory time-course of *Tmprss6* mRNA expression and p-Stat3 levels are different, indicating that p-Stat3 probably does not regulate *Tmprss6* mRNA expression.

**Figure 3:**
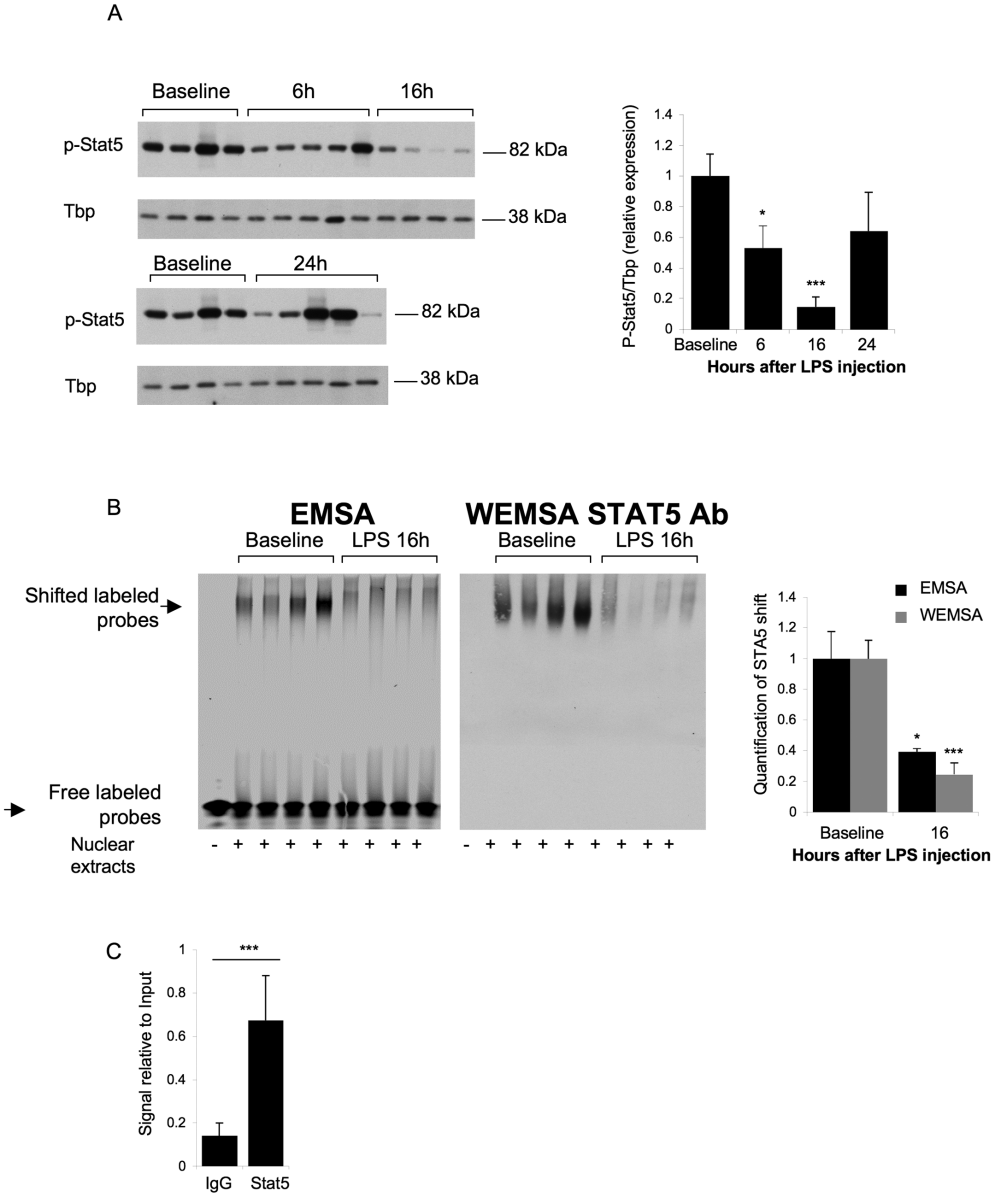
TMPRSS6 downregulation by inflammation is mediated by p-Stat5. (A) Liver nucleic lysates from mice were analyzed by Western blot for p-Stat5 relative to TBP. Values shown are means of expression values divided by a calibrator quantity (the mean value of expression for the baseline group)+/− SEM. Means in baseline and treated groups were compared by student t tests. (B) Hepatic nuclear extracts from mice at Baseline or 16 hrs after injection with LPS (n = 4 per group) were subjected to EMSA using a Stat5 binding sequence from the mouse *Tmprss6* promoter (left panel). Nucleic acids and bound proteins were transferred on membrane and analyzed by Western blot for Stat5. Stat5 shift was quantified using Li-Cor software. (C) Livers from Baseline mice (n = 5) were subjected to ChIP with Stat5 antibody and normal IgG antibody. Recovered DNA was analyzed for the presence of TMPRSS6 promoter. P values p<0.05 were considered statistically significant. Significances are: *p<0.05; ***p<0.005.

To further explore the mechanism of *Tmprss6* mRNA regulation by inflammation, we ran a computational analysis of the mouse *Tmprss6* gene promoter with Genomatix Software Suite. We identified one canonical Stat5 DNA-binding site (TTCN_3_GAA) in position −327 upstream of exon 1, conserved inter-species (Figure S4). We therefore evaluated the Stat5 phosphorylation level in liver nuclear extracts of wild-type mice injected with LPS. Injection of LPS induced a strong decrease of Stat5 phosphorylation 6 hours after LPS injection (by 50%), with a maximal decrease 16 hours after LPS injection by 87% (Figure 3A) compared to baseline mice, correlating with the decrease of *Tmprss6* mRNA expression.

To determine whether the *Tmprss6* gene promoter is a direct target of Stat5, we performed an electrophoretic mobility shift assay (EMSA) with a specific labeled probe containing the Stat5 element of the *Tmprss6* gene promoter. Compared to the migration of the free labeled probes in absence of nuclear extract (Figure 3B, left panel, lane 1), incubation with nuclear extracts from baseline mice induced a shift in migration, indicating protein binding on the Stat5 element of the *Tmprss6* gene promoter (Figure 3B, left panel, lane 2–5). Interestingly, 16 hours after LPS injection, when the Stat5 phosphorylation level was reduced (Figure 3A), the shift in migration was decreased by 60% (Figure 3B, left panel, lane 6–9). To confirm that Stat5 proteins were responsible for the shift in migration, we used a Combined Western Blotting Electrophoresis Mobility Shift Assay (WEMSA). After EMSA, bound proteins were transferred to a nitrocellulose membrane and immunoblotted with a STAT5 specific antibody. No signal was detected in the absence of nuclear extract (Figure 3B, right panel, lane 1). We were able to detect a single specific band at the same size as the shifted complex of the EMSA experiment (figure 3B, right panel). Moreover, the intensity of the signal corresponding to the binding of the STAT5 antibody was stronger when the nuclear extracts were prepared from liver of baseline mice (Figure 3B, right panel, lane 2–5) compared to nuclear extracts prepared from liver of mice injected with LPS for 16 hours (Figure 3B, right panel, lane 6–9). These results indicate that the EMSA complex contained Stat5 proteins bound to the Stat5 element on the *Tmprss6* promoter.

To test whether Stat5 recognizes this DNA-binding site in the *Tmprss6* promoter *in vivo*, we performed chromatin-immunoprecipitation (ChIP) experiments using Stat5 antibody on the liver of baseline mice. Quantitative real-time PCR analysis of immunoprecipitated Stat5-DNA complexes yielded the amplification of a PCR product representing Stat5-binding sites in the Tmprss6 promoter in specific Stat5 ChIP, but lower in control IgG ChIP experiments (Figure 3C).

To verify a functional role for Stat5 binding to the mouse *Tmprss6* promoter on *Tmprss6* mRNA expression, we studied the effect of Stat5 overexpression on *Tmprss6* promoter activity *in vitro* using a mouse *Tmprss6* promoter luciferase construct (*Tmprss6* wt-Luc) in a mouse hepatoma cell line (hepa 1–6 cells) since this construct was not functional in the human cell line Hep3B (data not shown). *Tmprss6 wt*-Luc was transfected in presence of an equal quantity of empty plasmid or construct encoding a mouse Stat5b (major form of STAT5 expressed in hepatocytes), and relative luciferase activity was measured 48 hours after transfection. Transfection of mStat5b led to a significant increase of total Stat5 protein (Figure S5A). mStat5b overexpression significantly increased *Tmprss6* promoter luciferase activity by 1.55 fold compared to empty vector (Figure 4A, black bars), confirming that Stat5 is a positive regulator of *Tmprss6* gene expression. Next, we mutated the palindromic half-site of the predicted mStat5 element on the *Tmprss6*-Luc construct (*Tmprss6* mt-Luc), a mutation that is sufficient to completely inhibit the binding of Stat5. This mutation reduced the increase of Tmprss6 promoter activity induced by mStat5b overexpression by 50% (Figure 4A, gray bars), indicating that Stat5 regulates Tmprss6 expression, at least in part, by directly binding its promoter. In addition to the binding of STAT5 on the perfect site TTCN_3_GAA, STAT5 transcription factors can also bind to related homologous sites such as the STAT6 binding sequence TTCN_4_GAA [19]. Indeed, we identified with the Genomatix software suite three Stat6 binding sequences in the mouse *Tmprss6* promoter that could explain the residual m*Tmprss6* promoter activity in response to Stat5 expression. *In vivo*, the non-binding of STAT5 transcription factor on the sequence TTCN_4_GAA is regulated by the chromatin conformation [19] that can be loosened on the *Tmprss6*-luc vector.

To further support the role of STAT5 in the regulation of *TMPRSS6* mRNA expression, we tested the effects of siRNA-mediated knockdown of STAT5b on *TMPRSS6* mRNA expression in Hep3B cells. Transfection with siRNA STAT5b decreased *STAT5b* mRNA expression by 67% (Figure S5B). Importantly, silencing *STAT5b* induced a significant downregulation of *TMPRSS6* mRNA expression by 30% compared to Hep3B cells transfected with siRNA control (Figure 4B). Together, these results reinforce the hypothesis that STAT5 is a positive regulator of TMPRSS6 expression. Interestingly, the silencing of *STAT5b* mRNA expression results also in a significant increase of HAMP mRNA expression by 1.25 fold (Figure S5C) similar to the increase induced by the silencing of *TMPRSS6* mRNA expression in non-treated cells (Figure 4C).

### Regulation of *TMPRSS6* Expression by Inflammation has a Functional Role in Hepcidin Modulation

To characterize the functional role of TMPRSS6 downregulation in the upregulation of hepcidin by inflammation, we transfected Hep3B cells with siRNA *TMPRSS6* [*10*] followed by treatment with 20 ng of IL-6 for 48 hours and measurement of *HAMP* mRNA expression. Forty-eight hours corresponds to a time point where *TMPRSS6* mRNA expression is no longer inhibited by IL-6 treatment (Figure S6). Inhibition of *TMPRSS6* mRNA expression allowed a significantly higher increase of *HAMP* mRNA expression in response to IL-6 treatment after 48 hours by 2-fold compared to Hep3B cells transfected with siRNA control (Figure 4C).

We next performed the converse experiment by transfecting Hep3B cells with human *HAMP* promoter/firefly luciferase reporter construct in the presence of an equal quantity of empty plasmid or a construct encoding human matriptase-2 [10]. Transfected Hep3B cells were treated with IL-6 for 16 hours and luciferase activity was measured. Treatment with IL-6 induced an increase of the *HAMP* promoter/firefly luciferase activity by 7.7 fold (Figure 4D). Over-expression of human matriptase-2 significantly prevented this increase and reduced the IL-6 mediated stimulation of *HAMP* promoter/firefly luciferase activity by 52% (Figure 4D). Together, these results indicate that, *in vitro*, the decrease of *TMPRSS6* mRNA expression induced by inflammation participates in the upregulation of hepcidin expression.

## Discussion

Inflammation due to infection, autoimmune disease, or cancer stimulates the production of many proinflammatory cytokines such as IL-6, leading to increased hepcidin expression. However, the antimicrobial and immune modulating properties of hepcidin need to be balanced with its role in controlling the availability of iron. Thus, hepcidin expression during inflammation needs to be finely regulated. Here, we identified a novel pathway for hepcidin regulation by inflammation via TMPRSS6 and STAT5 that is independent of the previously described STAT3 and BMP-SMAD pathways.

In this study, we demonstrated that treatment of a hepatoma cell line with IL-6 or injection of IL-6 or LPS in mice decreased *TMPRSS6* mRNA expression. The reduced potency of IL-6 compared with LPS to suppress TMPRSS6 mRNA suggests that LPS may regulate TMPRSS6 through other mechanisms in addition to IL-6, similar what has been reported for hepcidin [15]. We also characterized the mechanism of TMPRSS6 regulation by inflammation. We provided several lines of evidence showing that inflammation downregulates *Tmprss6* via decreasing Stat5 phosphorylation. STAT5a/b are transcription factors regulated by a wide variety of cytokines such as interleukins. Importantly, consistent with the results in our study, published literature shows that in response to turpentine-induced inflammation in rats, Stat5b protein level is reduced in the nucleus of the liver 12 hours after injection [20]. The mechanism(s) by which inflammatory cytokines decrease Stat5 signaling remain to be elucidated.

Global *Stat5a/b^−/−^* and hematopoietic-specific *Stat5a/b^−/−^* mice have a severe microcytic hypochromic anemia that is proposed to be related to the lack of Stat5 binding to the promoter of *Irp2* and *Tfr1,* leading to the inhibition of iron uptake in erythroid cells [21]. Interestingly, global *Stat5a/b^−/−^* mice have a more severe microcytic hypochromic anemia than hematopoietic-specific *Stat5a/b^−/−^* mice, suggesting that the role of Stat5 in other cell types may contribute to the severity of the microcytic hypochromic anemia. Indeed, Stat5 seems to regulate the transcription of many other genes involved in iron metabolism, including TMPRSS6 as demonstrated in our study. Conditional knockout of Stat5 in other cell types including hepatocytes will be relevant to characterize a more complete role of Stat5 in iron metabolism.

*TMPRSS6* expression is stimulated by EPO, [9] that is a positive regulator of STAT5-phosphorylation [22] and a negative regulator of hepcidin expression [23]. Since we identified in this study that Stat5 is a positive regulator of *Tmprss6* expression, it can be hypothesize that an upregulation of STAT5-phosphorylation and consequent TMPRSS6 induction by EPO injection may play a role in the regulatory mechanism leading to the hepcidin decrease in response to EPO. Further studies will be required to address this possible role.

In a recent study published by Nai et al [24], the authors highlighted that in a mouse model of β-thalassemia (Hbb^th3/+^ mice), serum EPO level is elevated, *Hamp* mRNA expression in the liver is decreased, and *Id1* and *Tmprss6* mRNA expression in the liver are upregulated. Since ID1 is a positive regulator of TMPRSS6 expression [10], the authors suggested that the upregulation of *Tmprss6* mRNA expression seen in Hbb^th3/+^ mice is induced by the upregulation of Id1 expression. Here, we demonstrated that in addition to ID1, STAT5 is also a positive regulator of TMPRSS6 expression. Since EPO is increased in Hbb^th3/+^ mice, we hypothesize that an upregulation of Stat5-phophorylation by EPO could also be involved in *Tmprss6* upregulation seen in Hbb^th3/+^ mice.

**Figure 5:**
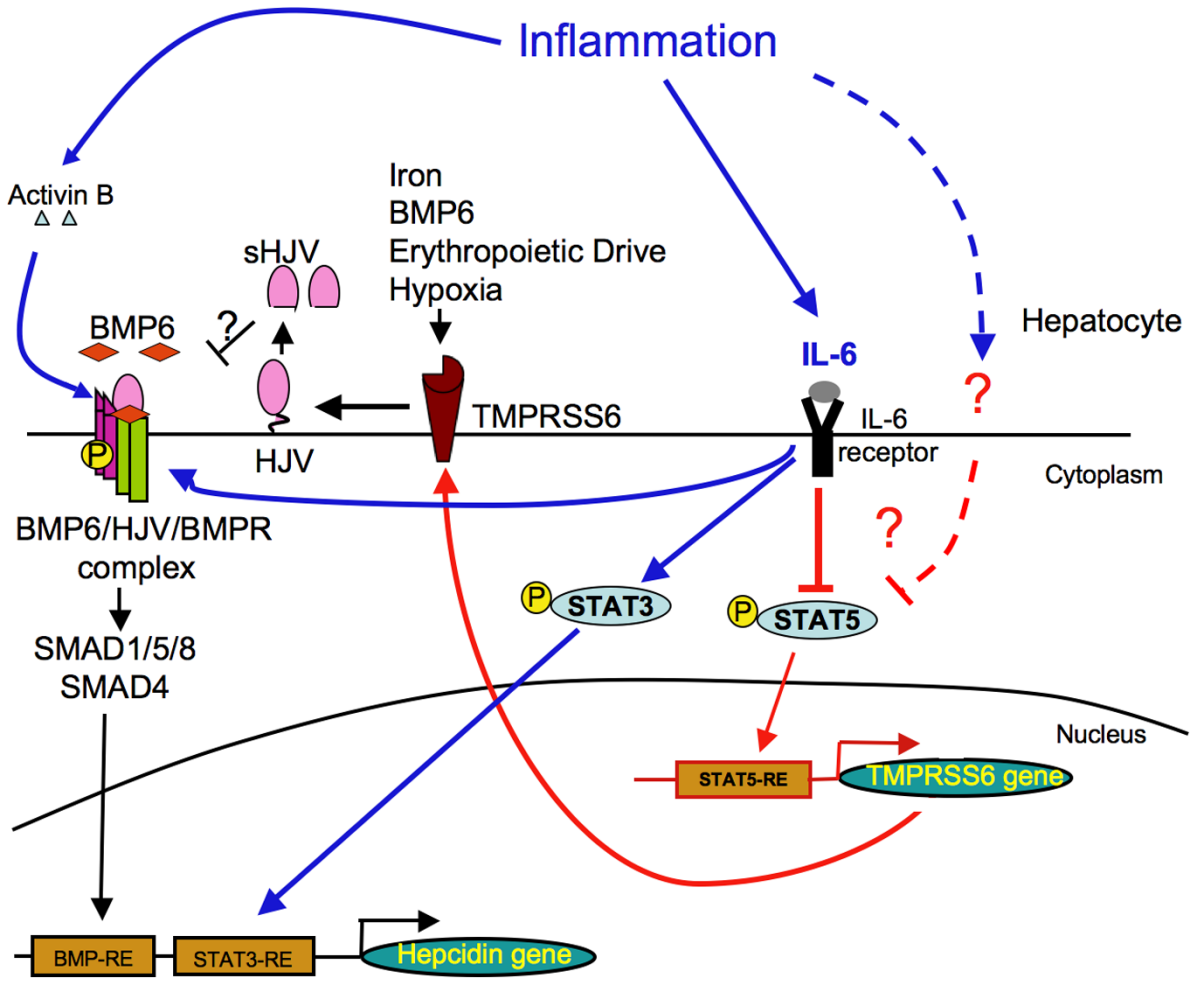
Schematic representation showing proposed role of TMPRSS6 regulation by inflammation via STAT5. We propose that in addition to being stimulated by several signals that regulate hepcidin such as iron, BMP6, erythropoeitic drive, and hypoxia, TMPRSS6 expression is also inhibited indirectly by IL-6. IL-6 stimulates hepcidin expression through the induction of STAT3 phosphorylation and through the stimulation of the BMP6-HJV-SMAD pathway activity, leading to binding of p-STAT3 on STAT3-responsive element (STAT3-RE) and SMAD complexes to BMP-responsive elements (BMP-REs) on the hepcidin promoter. In parallel, inflammation through IL-6 and/or through another non-identified cytokine, inhibits the STAT5 phosphorylation leading to a decrease of P-STAT5 binding on the STAT5-responsive element (STAT5-RE) of the TMPRSS6 promoter resulting in a decrease of TMPRSS6 expression. TMPRSS6 is a negative feedback inhibitor of BMP-SMAD pathway activity and hepcidin expression by cleaving the BMP co-receptor HJV. By inhibiting this negative feedback, TMPRSS6 participates to the hepcidin increase in response to IL-6.

Our results suggest that inflammation regulates TMPRSS6 expression, and in turn TMPRSS6 has a functional role on hepcidin regulation under inflammation conditions. Hepcidin regulation by inflammation is known to occur through the IL6/STAT3 [13] pathway but also through the BMP-SMAD pathway independently of IL-6 via the regulation of activin B [15]. This current work highlights an additional regulatory mechanism through IL-6 involving matriptase-2 (Figure 5). Determining what is the exact responsibility *in vivo* of each regulatory mechanism involved in hepcidin modulation by inflammation is challenging since both STAT3 and BMP-SMAD pathway are required [14]. However, it is clear that all of these mechanisms have the ability to independently regulate hepcidin expression [12], [13], [15]. In our LPS injected mice cohort, the hepcidin level peaks after 6 hrs, whereas activin B peaks at 4 hrs (Figure 1 and Figure S7). Although TMPRSS6 is maximally decreased after 16 hrs, it is already significantly decreased by 6 hrs, and in fact levels at 16 hrs were not significantly different from 6 hrs. In addition, LPS induces the highest acute STAT3 phosphorylation before the hepcidin peak [15]. The fact that none of the known regulatory mechanism has exactly the same time-course than hepcidin in response to inflammation leads to the hypothesis that the hepcidin level peak may results from the combination of all the regulatory mechanisms at 6 hrs after LPS injection (i.e. low TMPRSS6 level, high STAT3-phosphorylation, and high activin B level). Indeed, this multi-layered regulatory mechanism is consistent with a fine-tuning of hepcidin expression in response to inflammation.

Chronic inflammation and the consequent increase of hepcidin are responsible for anemia of chronic diseases (ACD) [25]. Currently available treatment strategies for ACD have limited success and can increase risk of infections, mortality, iron overload [26]. New therapeutic strategies are currently being developed in order to target the hepcidin axis and decrease hepcidin production such as BMP-SMAD pathway inhibitors [27]. Since we have demonstrated that TMPRSS6 expression is downregulated by inflammation, and TMPRSS6 is a known inhibitor of hepcidin expression, our results suggest that stimulating TMPRSS6 expression in patients with ACD could be another approach to prevent the hepcidin over-expression seen in ACD.

In summary, we demonstrate here that *TMPRSS6* is regulated by inflammation and has a functional role in regulating hepcidin expression in this setting (Figure 5). We also show for the first time that in addition to its role on Irp2 and Tfr1 expression, Stat5 regulates Tmprss6 (Figure 5), emphasizing the important role of Stat5 in iron metabolism. Understanding the mechanism and the role of inflammation on TMPRSS6 regulation may ultimately lead to new therapeutic strategies to treat diseases where hepcidin levels are deregulated such as β-thalassemia and anemia of chronic disease.

## Supporting Information

Figure S1 (A–B) HepG2 cells were treated with 100 ng/mL of IL-6 for 16 hours and were analyzed for *HAMP* and *TMPRSS6* relative to *RPL19* mRNA expression by quantitative real-time RT- PCR. For each experiment, raw data were normalized to the expression value in the control group. Values shown are means of normalized expression values in 4 independent experiments+/− SEM. Means in IL-6 treated cells were compared to one by one sample student t tests. *p<0.05.
(TIF)

Figure S2 Eight-week-old male C57BL/6 mice received one intraperitoneal injection of LPS 1 µg/g body weight (n = 5 per group) and were sacrificed 6, 16 and 24 hours after injection. *Crp* and *Il-6* relative to *Rpl19* mRNA expression were analyzed by quantitative real-time RT- PCR. Values shown are means of expression values divided by a calibrator quantity (the mean value of expression for the baseline group)+/− SEM. Means in baseline and treated groups were compared by student t tests. ***p<0.005; ****p<0.001.
(TIF)

Figure S3 Liver nucleic lysates from baseline mice and mice injected with LPS were used to measure the p-Stat3 protein level. 4 µg of proteins were subjected to western-blot analysis with rabbit anti-p-Stat3 (1∶1000, Cell Signaling). Membrane was stripped with and reprobed with TBP antibody (1/1000). Values shown are means of expression values divided by a calibrator quantity (the mean value of expression for the baseline group)+/− SEM. Means in baseline and treated groups were compared by student t tests. ***p<0.005; ****p<0.001.
(TIF)

Figure S4 Promoter analysis for transcription binding sites was run with Genomatix Software Suite and indicate the presence of STAT5 binding sequence in the TMPRSS6 promoter in Mouse, Rat and Human.
(TIF)

Figure S5 (A) Cell lysates prepared with 1X passive lysis buffer for the luciferase experiment were used to measure the Stat5 protein level. 10 µg of proteins were subjected to western-blot analysis with rabbit anti-Stat5 (1∶1000, Santa-cruz). Membrane was stripped with and reprobed with Actin antibody (1/10000) (B,C) Hep3B cells were reverse-transfected with 10 nM of control siRNA or human STAT5b. Five hours later, the transfection media was replaced with culture medium to stop the transfection. Twenty-four hours later, cells were serum starved with FBS 1% medium then harvested for RNA extraction 24 hours later. Stat5b and *HAMP* relative to *RPL19* mRNA expression were analyzed by quantitative real-time RT- PCR. Values shown are means of expression values divided by a calibrator quantity (the mean value of expression for the mock)+/− SEM. Means in siRNA control and siRNA STAT5b groups were compared by student t tests. *p<0.05; ****p<0.001.
(TIF)

Figure S6 Hep3B cells were treated with 20 ng/mL of IL-6 for several time points between 1 and 48 hours and were analyzed for *TMPRSS6* relative to *RPL19* mRNA expression by quantitative real-time RT- PCR. For each experiment, raw data were normalized to the expression value of the non-treated cells. Values shown are means of normalized expression values in 6 independent experiments+/− SEM. Means in IL-6 treated groups were compared to 1 by one-sample student t tests. *p<0.05.
(TIF)

Figure S7 (A) Eight-week-old male C57BL/6 mice received one intraperitoneal injection of LPS 1 µg/g body weight (n = 5 per group) and were sacrificed 4, 6, 16 and 24 hours after injection. *Inhbb* relative to *Rpl19* mRNA expression was analyzed by quantitative real-time RT- PCR. Values shown are means of expression values divided by a calibrator quantity (the mean value of expression for the baseline group)+/− SEM. Means in baseline and treated groups were compared by student t tests. (B,C) Eight-week-old male C57BL/6 mice received one intraperitoneal injection of LPS 1 µg/g body weight (n = 5 per group) and were sacrificed 4 hours after injection. *Hamp* and *Tmprss6* relative to *Rpl19* mRNA expression were analyzed by quantitative real-time RT- PCR. Values shown are means of expression values divided by a calibrator quantity (the mean value of expression for the baseline group)+/− SEM. Means in baseline and treated groups were compared by student t tests. **p<0.01; ****p<0.001.
(TIF)

Table S1 Primer sequences.
(TIF)
