# Supplementary figures and images for: Inflammation Regulates TMPRSS6 Expression via STAT5
Source: PLoS One. 2013 Dec 23;8(12):e82127. doi: 10.1371/journal.pone.0082127 (PMC3871639; doi:10.1371/journal.pone.0082127)

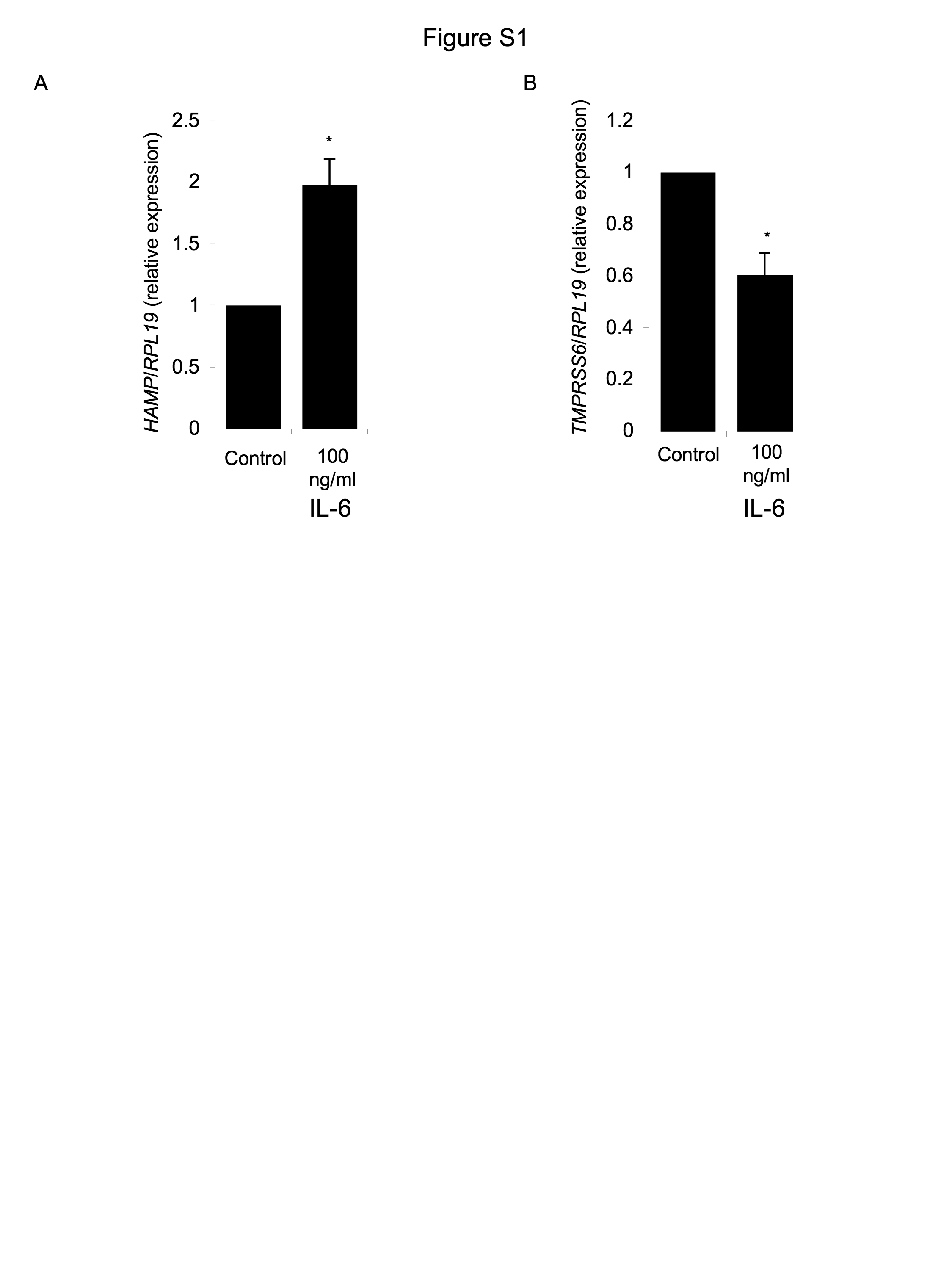

Supplement: Figure S1 — (A–B) HepG2 cells were treated with 100 ng/mL of IL-6 for 16 hours and were analyzed for HAMP and TMPRSS6 relative to RPL19 mRNA expression by quantitative real-time RT- PCR. For each experiment, raw data were normalized to the expression value in the control group. Values shown are means of normalized expression values in 4 independent experiments+/− SEM. Means in IL-6 treated cells were compared to one by one sample student t tests. *p<0.05. (TIF) [file pone.0082127.s001.tif]

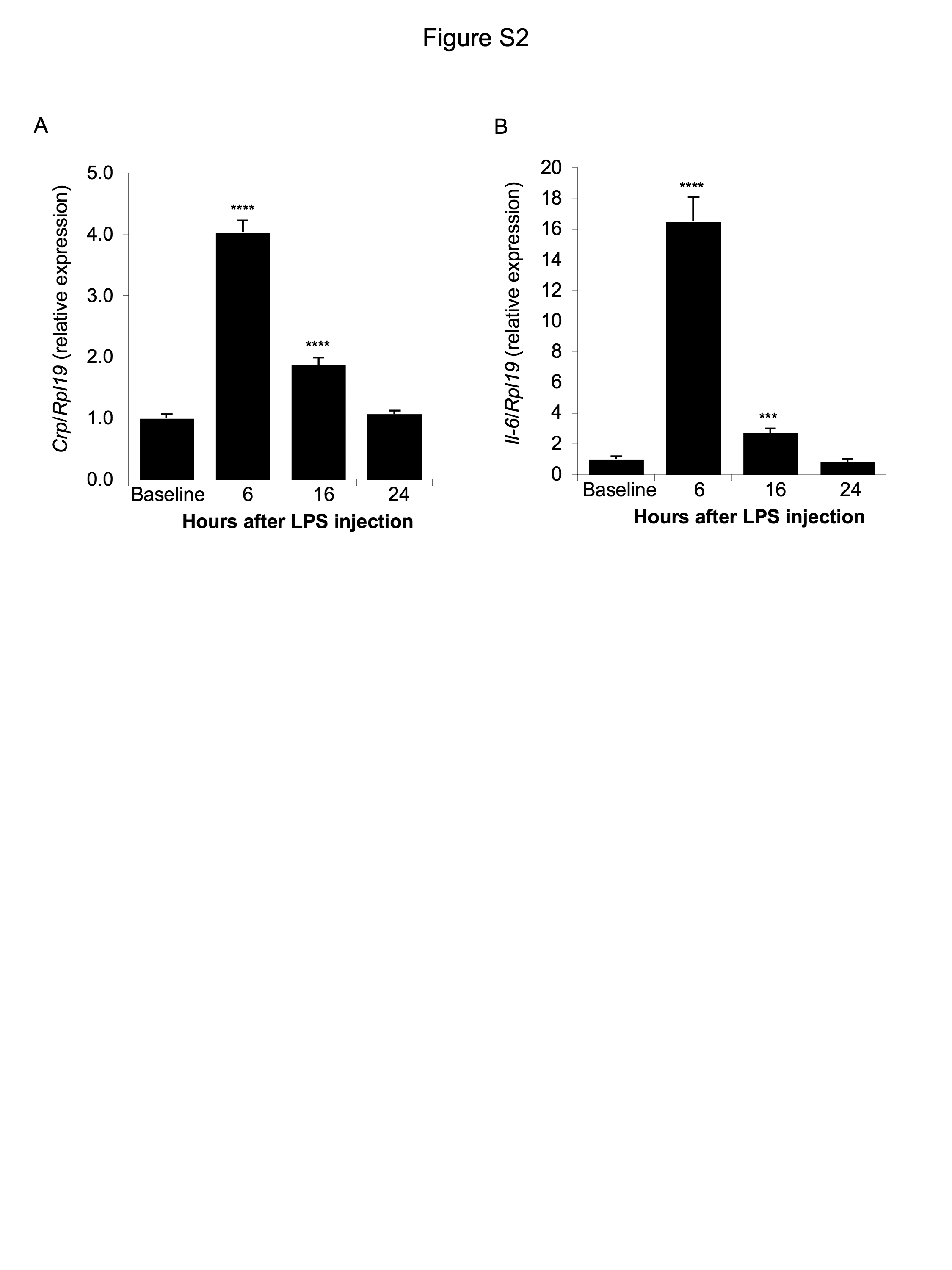

Supplement: Figure S2 — Eight-week-old male C57BL/6 mice received one intraperitoneal injection of LPS 1 µg/g body weight (n = 5 per group) and were sacrificed 6, 16 and 24 hours after injection. Crp and Il-6 relative to Rpl19 mRNA expression were analyzed by quantitative real-time RT- PCR. Values shown are means of expression values divided by a calibrator quantity (the mean value of expression for the baseline group)+/− SEM. Means in baseline and treated groups were compared by student t tests. ***p<0.005; ****p<0.001. (TIF) [file pone.0082127.s002.tif]

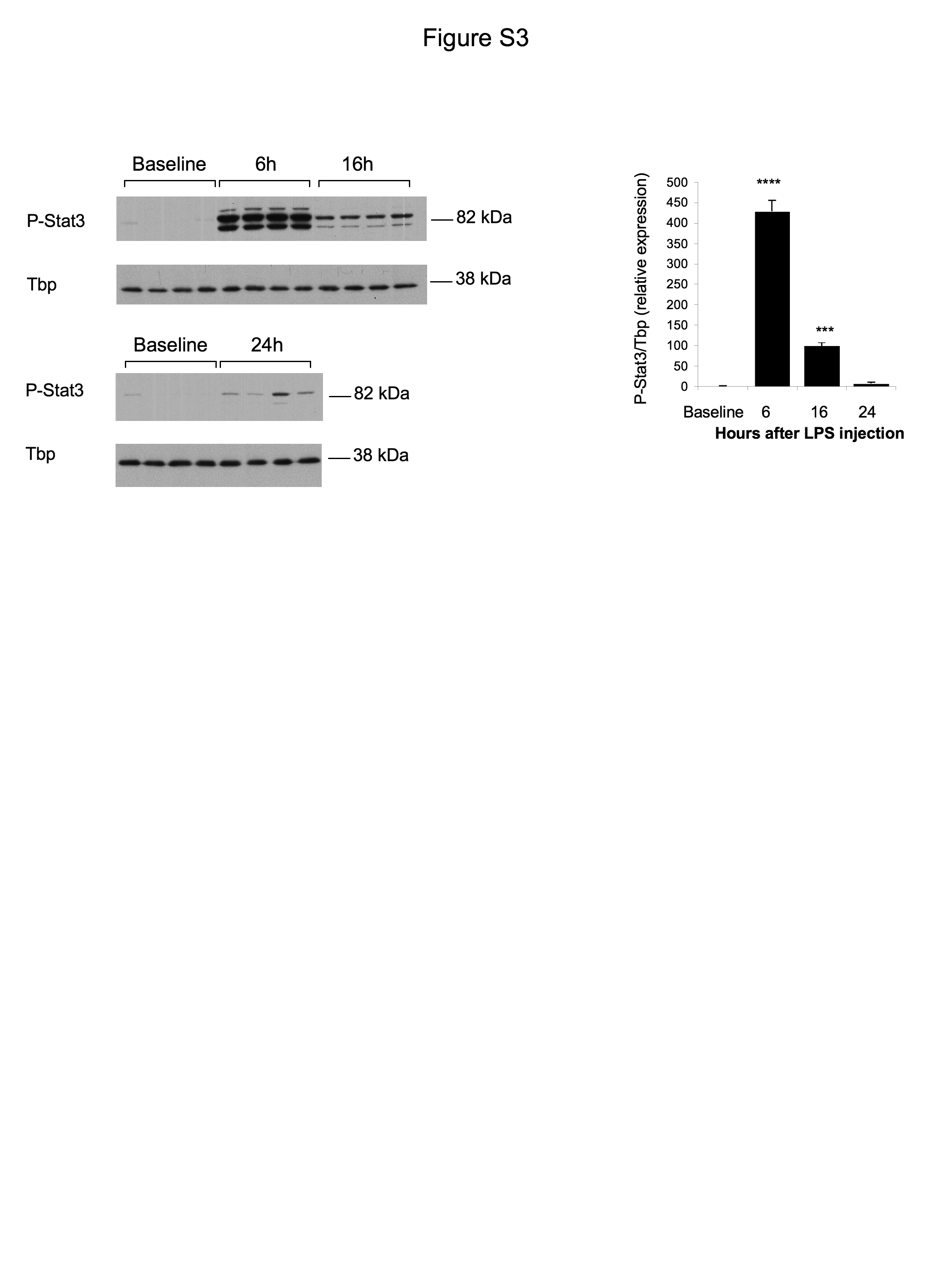

Supplement: Figure S3 — Liver nucleic lysates from baseline mice and mice injected with LPS were used to measure the p-Stat3 protein level. 4 µg of proteins were subjected to western-blot analysis with rabbit anti-p-Stat3 (1∶1000, Cell Signaling). Membrane was stripped with and reprobed with TBP antibody (1/1000). Values shown are means of expression values divided by a calibrator quantity (the mean value of expression for the baseline group)+/− SEM. Means in baseline and treated groups were compared by student t tests. ***p<0.005; ****p<0.001. (TIF) [file pone.0082127.s003.tif]

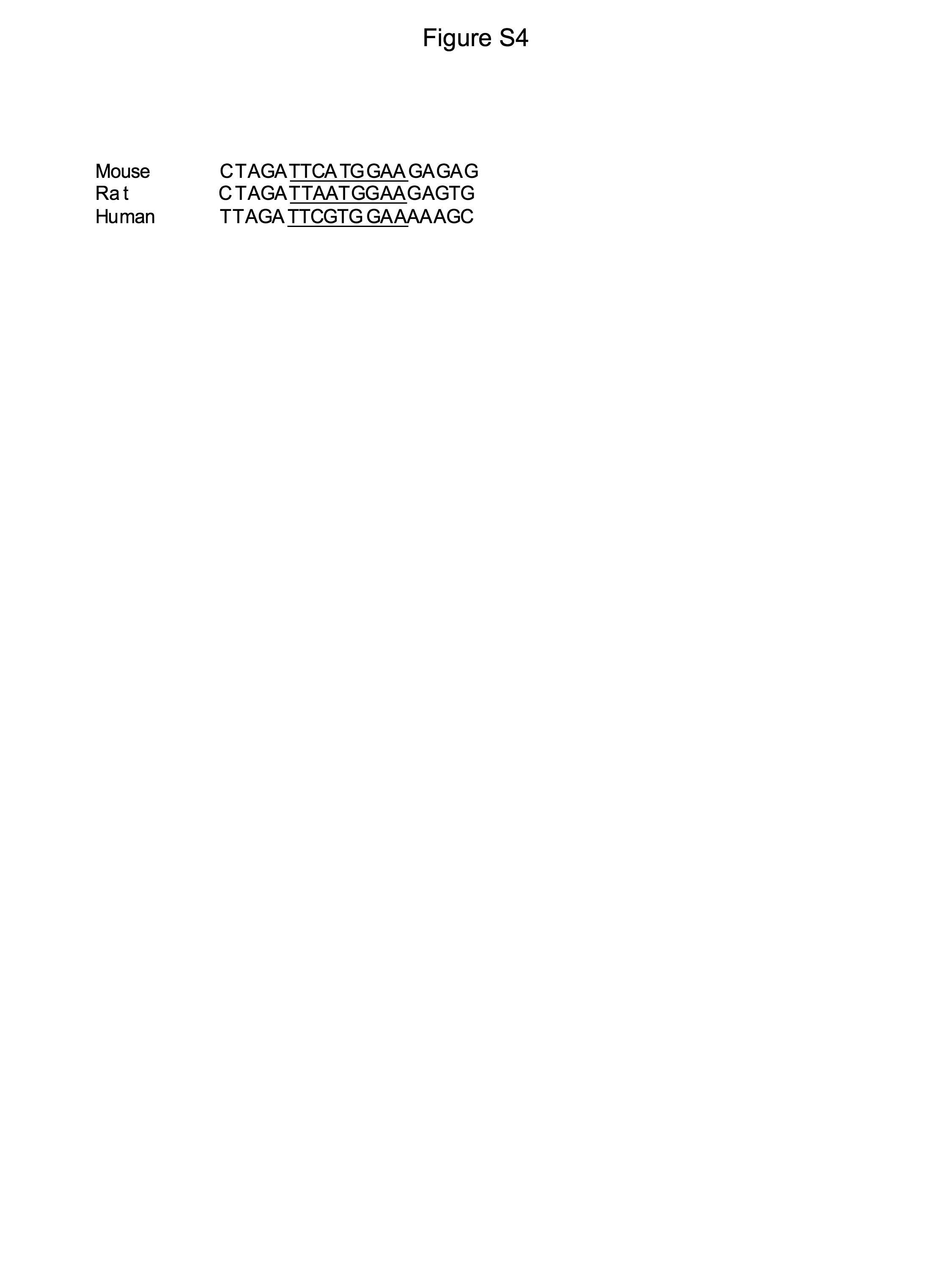

Supplement: Figure S4 — Promoter analysis for transcription binding sites was run with Genomatix Software Suite and indicate the presence of STAT5 binding sequence in the TMPRSS6 promoter in Mouse, Rat and Human. (TIF) [file pone.0082127.s004.tif]

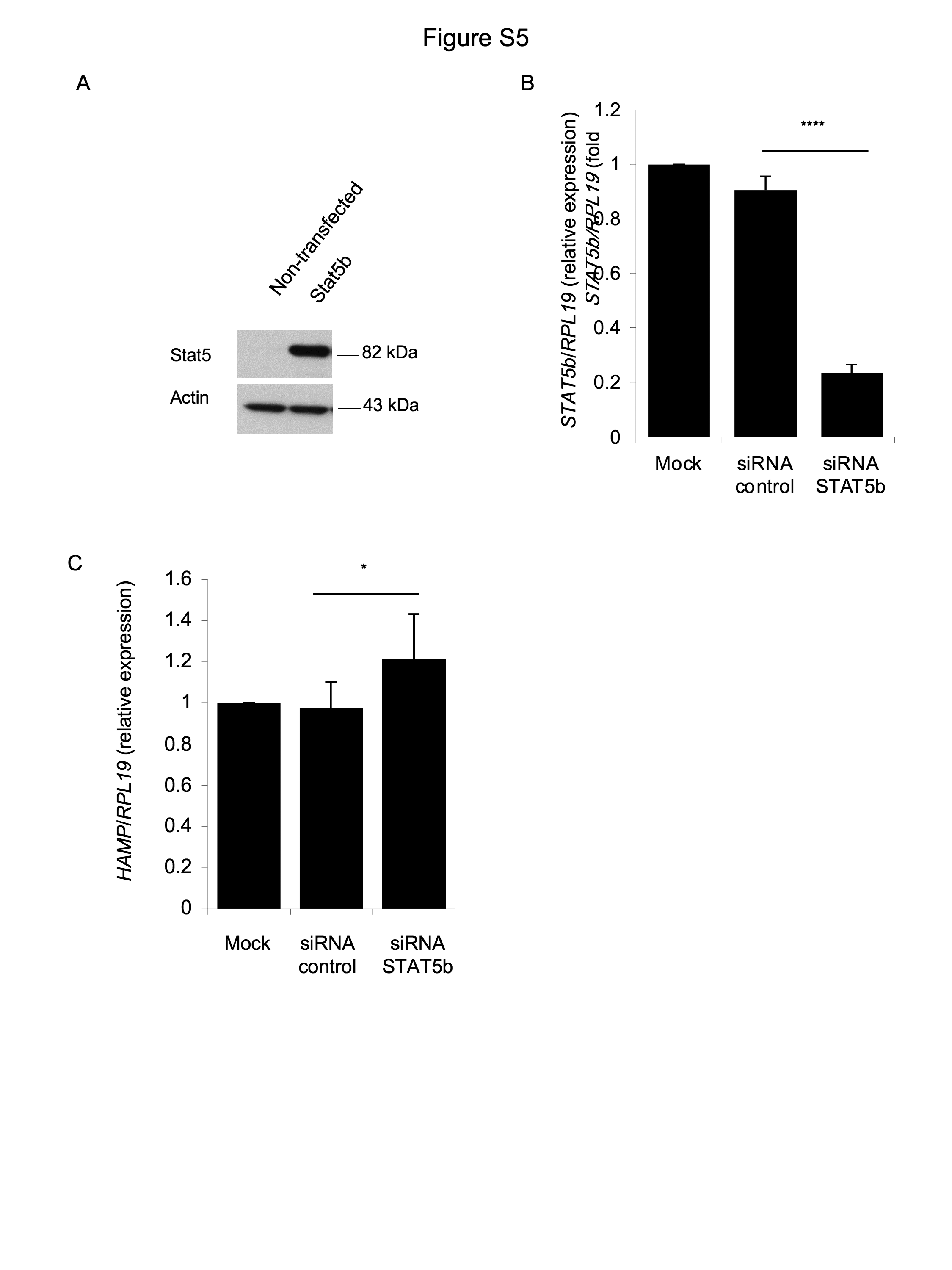

Supplement: Figure S5 — (A) Cell lysates prepared with 1X passive lysis buffer for the luciferase experiment were used to measure the Stat5 protein level. 10 µg of proteins were subjected to western-blot analysis with rabbit anti-Stat5 (1∶1000, Santa-cruz). Membrane was stripped with and reprobed with Actin antibody (1/10000) (B,C) Hep3B cells were reverse-transfected with 10 nM of control siRNA or human STAT5b. Five hours later, the transfection media was replaced with culture medium to stop the transfection. Twenty-four hours later, cells were serum starved with FBS 1% medium then harvested for RNA extraction 24 hours later. Stat5b and HAMP relative to RPL19 mRNA expression were analyzed by quantitative real-time RT- PCR. Values shown are means of expression values divided by a calibrator quantity (the mean value of expression for the mock)+/− SEM. Means in siRNA control and siRNA STAT5b groups were compared by student t tests. *p<0.05; ****p<0.001. (TIF) [file pone.0082127.s005.tif]

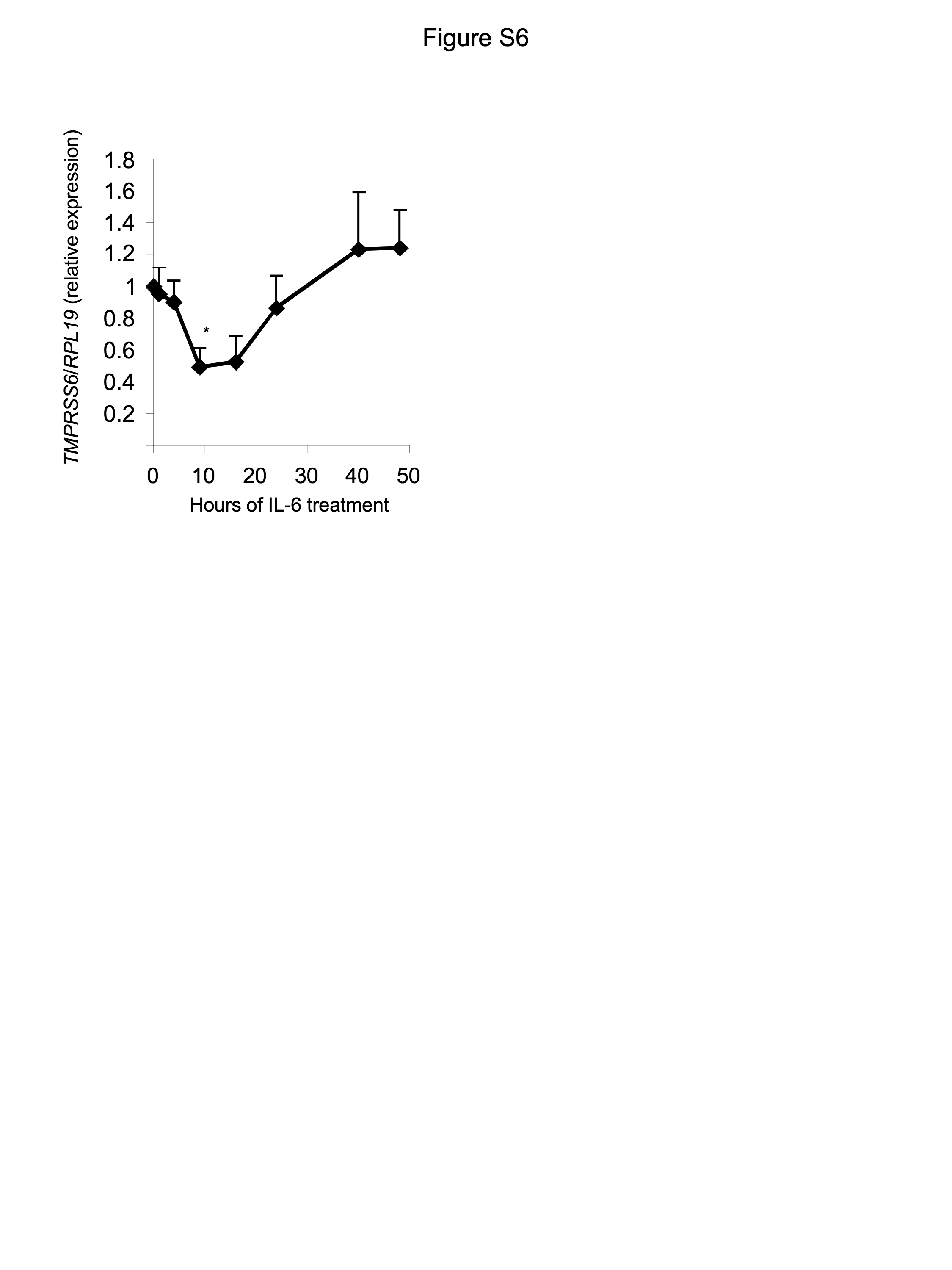

Supplement: Figure S6 — Hep3B cells were treated with 20 ng/mL of IL-6 for several time points between 1 and 48 hours and were analyzed for TMPRSS6 relative to RPL19 mRNA expression by quantitative real-time RT- PCR. For each experiment, raw data were normalized to the expression value of the non-treated cells. Values shown are means of normalized expression values in 6 independent experiments+/− SEM. Means in IL-6 treated groups were compared to 1 by one-sample student t tests. *p<0.05. (TIF) [file pone.0082127.s006.tif]

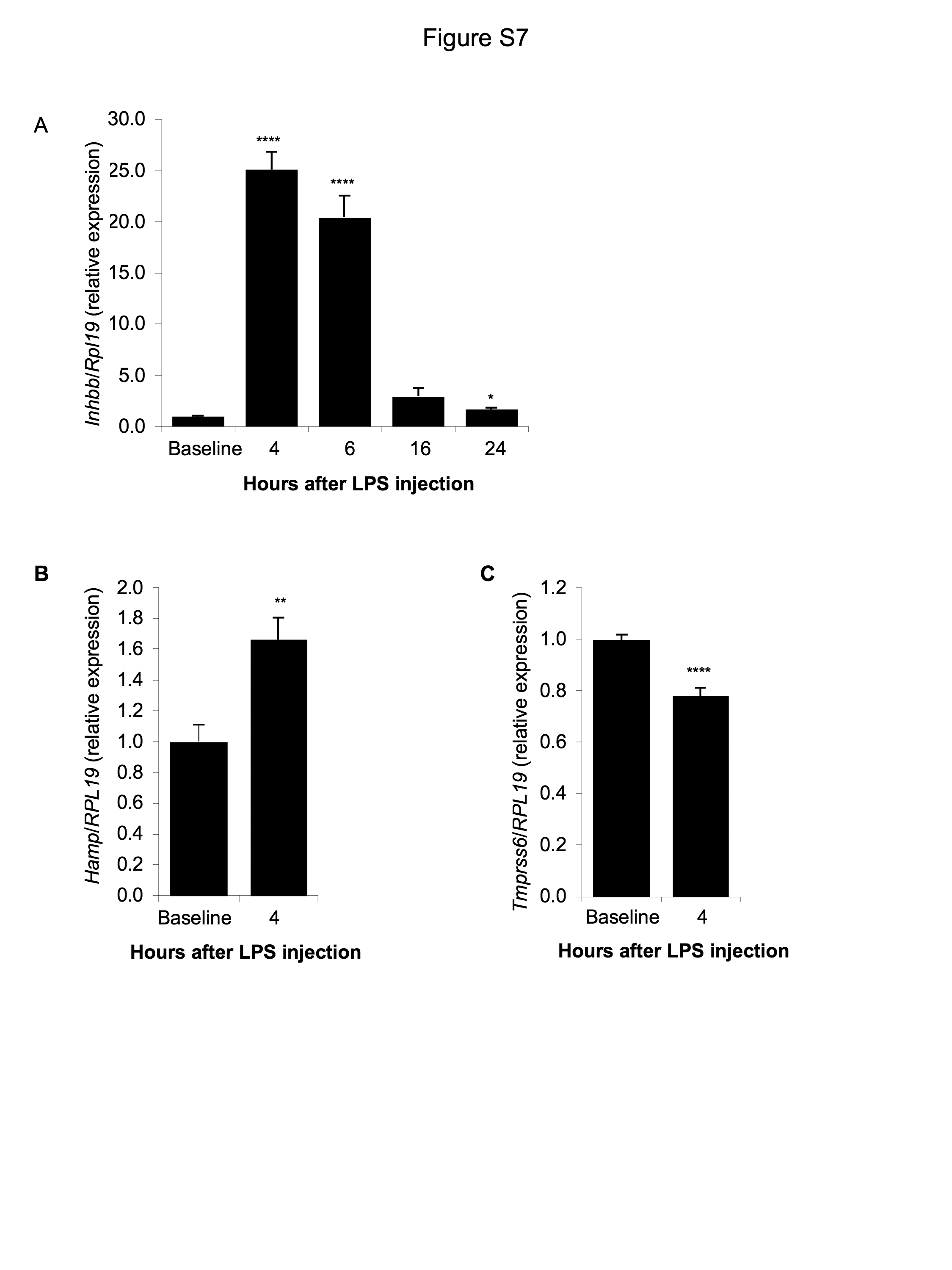

Supplement: Figure S7 — (A) Eight-week-old male C57BL/6 mice received one intraperitoneal injection of LPS 1 µg/g body weight (n = 5 per group) and were sacrificed 4, 6, 16 and 24 hours after injection. Inhbb relative to Rpl19 mRNA expression was analyzed by quantitative real-time RT- PCR. Values shown are means of expression values divided by a calibrator quantity (the mean value of expression for the baseline group)+/− SEM. Means in baseline and treated groups were compared by student t tests. (B,C) Eight-week-old male C57BL/6 mice received one intraperitoneal injection of LPS 1 µg/g body weight (n = 5 per group) and were sacrificed 4 hours after injection. Hamp and Tmprss6 relative to Rpl19 mRNA expression were analyzed by quantitative real-time RT- PCR. Values shown are means of expression values divided by a calibrator quantity (the mean value of expression for the baseline group)+/− SEM. Means in baseline and treated groups were compared by student t tests. **p<0.01; ****p<0.001. (TIF) [file pone.0082127.s007.tif]

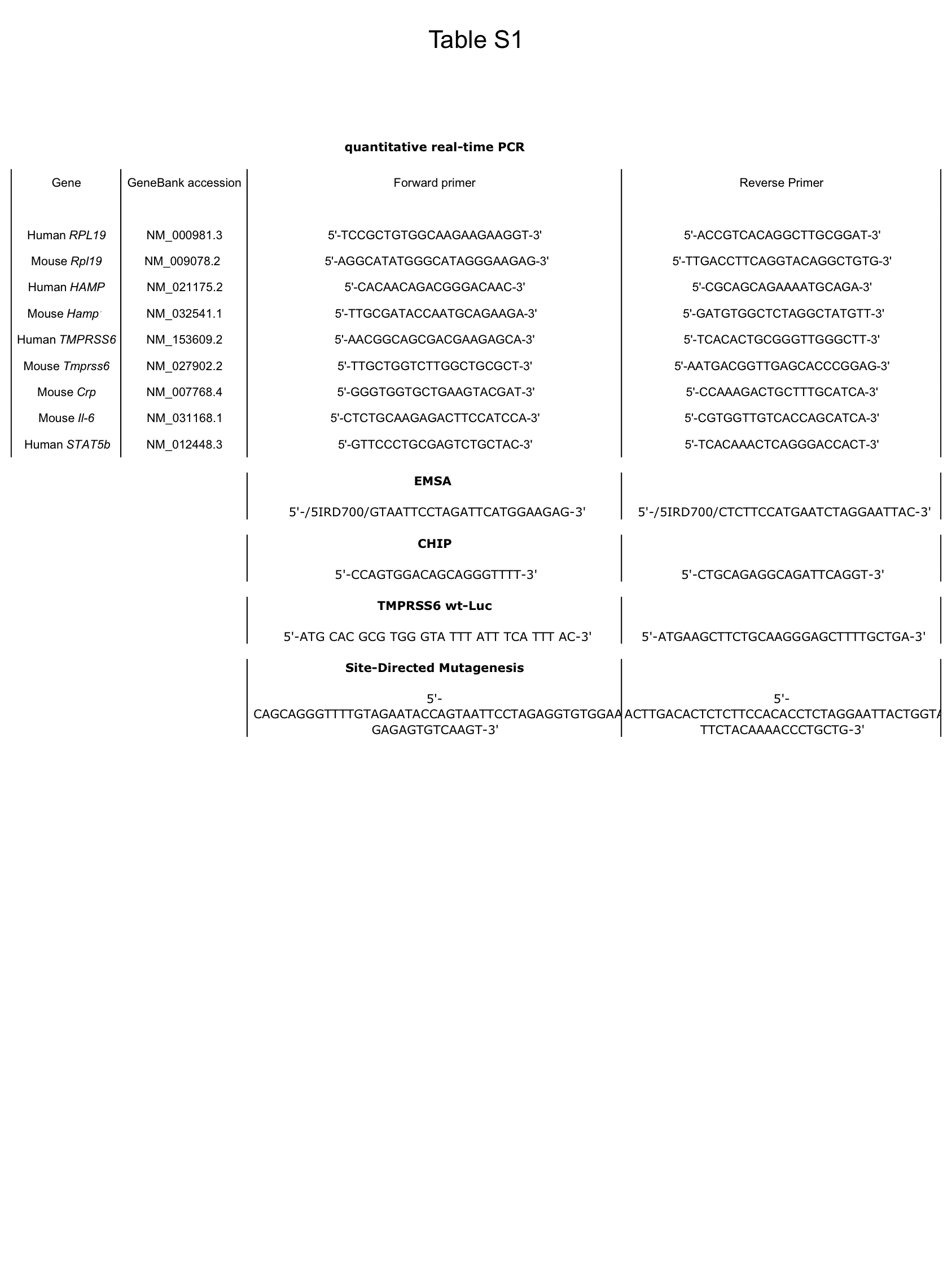

Supplement: Table S1 — Primer sequences. (TIF) [file pone.0082127.s008.tif]
